# Amyloid Beta and Phosphorylated Tau-Induced Defective Autophagy and Mitophagy in Alzheimer’s Disease

**DOI:** 10.3390/cells8050488

**Published:** 2019-05-22

**Authors:** P. Hemachandra Reddy, Darryll MA Oliver

**Affiliations:** 1 Internal Medicine Department, Texas Tech University Health Sciences Center, 3601 4th Street, Lubbock, TX 79430, USA; darryll.oliver@ttuhsc.edu; 2 Garrison Institute on Aging, Texas Tech University Health Sciences Center, 3601 4th Street, Lubbock, TX 79430, USA; 3 Garrison Institute on Aging, South West Campus, Texas Tech University Health Sciences Center, 6630 S. Quaker Suite E, Lubbock, TX 79413, USA; 4 Pharmacology & Neuroscience Department, Texas Tech University Health Sciences Center, 3601 4th Street, Lubbock, TX 79430, USA; 5 Cell Biology and Biochemistry, Texas Tech University Health Sciences Center, 3601 4th Street, Lubbock, TX 79430, USA; 6 Neurology Department, Texas Tech University Health Sciences Center, 3601 4th Street, Lubbock, TX 79430, USA; 7 Speech, Language and Hearing Sciences Department, Texas Tech University Health Sciences Center, 3601 4th Street, MS 9424, Lubbock, TX 79430, USA; 8 Department of Public Health, Graduate School of Biomedical Sciences, 3601 4th Street, Lubbock, TX 79430, USA

**Keywords:** Alzheimer’s disease, amyloid beta, phosphorylated tau, mitochondria and reactive oxygen species

## Abstract

Alzheimer’s disease (AD) is a progressive neurodegenerative disease characterized by memory loss and multiple cognitive impairments. Several decades of intense research have revealed that multiple cellular changes are implicated in the development and progression of AD, including mitochondrial damage, synaptic dysfunction, amyloid beta (Aβ) formation and accumulation, hyperphosphorylated tau (P-Tau) formation and accumulation, deregulated microRNAs, synaptic damage, and neuronal loss in patients with AD. Among these, mitochondrial dysfunction and synaptic damage are early events in the disease process. Recent research also revealed that Aβ and P-Tau-induced defective autophagy and mitophagy are prominent events in AD pathogenesis. Age-dependent increased levels of Aβ and P-Tau reduced levels of several autophagy and mitophagy proteins. In addition, abnormal interactions between (1) Aβ and mitochondrial fission protein Drp1; (2) P-Tau and Drp1; and (3) Aβ and PINK1/parkin lead to an inability to clear damaged mitochondria and other cellular debris from neurons. These events occur selectively in affected AD neurons. The purpose of our article is to highlight recent developments of a Aβ and P-Tau-induced defective autophagy and mitophagy in AD. This article also summarizes several aspects of mitochondrial dysfunction, including abnormal mitochondrial dynamics (increased fission and reduced fusion), defective mitochondrial biogenesis, reduced ATP, increased free radicals and lipid peroxidation, and decreased cytochrome *c* oxidase (COX) activity and calcium dyshomeostasis in AD pathogenesis. Our article also discusses how reduced levels of Drp1, Aβ, and P-Tau can enhance the clearance of damaged mitochondria and other cellular debris by autophagy and mitophagy mechanisms.

## 1. Alzheimer’s Disease

Alzheimer’s disease (AD) is an end-of-life neurodegenerative disease characterized by the presence of amyloid beta (Aβ) peptides (which collect to form senile plaques), as well as the formation and accumulation of hyperphosphorylated tau and neurofibrillary tangles (NFTs) in the brains of AD patients [1,2,3,4,5,6,7]. AD has had a major impact on the global economy, costing $800 billion in 2015 [3]. This cost is expected to increase each year, as the number of cases of AD is expected to rise from ~50 million at present to 82 million by 2030, and 152 million by 2050 [4]. The pathological features of AD include synaptic dysfunction, resultant cognitive impairment, and short-term memory loss, where persons are unable to formulate new memories [4,5].

The disease is believed to originate with neuronal degeneration in the second layer of the entorhinal cortex, and progresses to the hippocampus, temporal cortex, front-parietal cortex, and further to the subcortical nuclei [4,8]. AD brains and cerebrospinal fluid are noted to have elevated levels of malondialdehyde (MA) and 4-hydroxy nonenal (4-HNE). Several years of intense research have revealed that multiple cellular changes are implicated in the development and progression of disease, including mitochondrial damage, synaptic damage, Aβ formation and accumulation, inflammatory responses, the formation and accumulation of hyperphosphorylated tau (P-Tau) and NFTs, hormonal imbalance, and neuronal loss [5,6,8,9,10]. Among these cellular changes, mitochondrial dysfunction and synaptic damage are early changes in AD pathogenesis [6].

**Figure 1:**
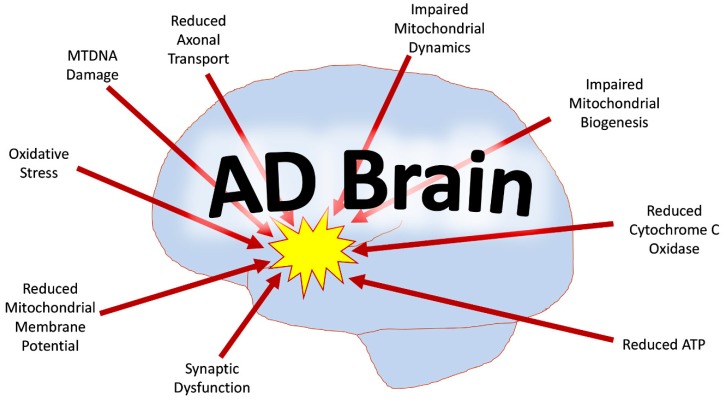
Mitochondrial abnormalities in the Alzheimer’s disease (AD) brain. Mitochondrial dysfunction in the AD brain includes mtDNA damage, reduced mtDNA copies, increased oxidative damage, reduced mitochondrial axonal transport, reduced mitochondrial membrane potential, dysfunctional mtDNA expression, rise in mtDNA mutations, impaired mitochondrial dynamics, and defective mitochondrial biogenesis.

Mitochondrial dysfunction in AD includes mtDNA damage, dysfunctional mtDNA expression, increased mtDNA mutations, reduced mtDNA copies, increased oxidative damage, reduced mitochondrial axonal transport, and overall impaired mitochondrial dynamics (Figure 1) [11,12,13]. AD likely causes the inhibition of Aβ clearance, and thus the Aβ accumulates in neurons [2]. Aβ induces much of the mitochondrial dysfunction, including oxidative stress, which contributes to phosphorylation of tau, mtDNA damage, and further Aβ interaction with Drp1, Aβ-binding alcohol dehydrogenase (ABAD) and CypD, loss of cytochrome *c* oxidase (COX) activity, impaired gating of the mitochondrial permeability transition pore, loss of membrane potential, and loss of cardiolipins [2,14]. The mitochondria themselves tend to be greater in number and smaller in size due to excessive mitochondrial fragmentation, especially in the presence of Aβ and P-Tau [2,4,7,14,15]. As mitochondrial dysfunction persists, mitochondrial biogenesis declines, reducing the ATP, which provides energy for synaptic vesicles to deliver neurotransmitters to the synapse [16]. This in turn also contributes to synaptic dysfunction [16].

The loss of COX and the resultant apoptotic pathway triggers the loss of neurons in the central nervous system (CNS) [2]. Normally, cardiolipin lost from the inner mitochondrial membrane (IMM) relocates to the surface of the outer mitochondrial membrane (OMM) and interacts with microtubule-associated protein 1A/1B-light chain 3 (LC3) to activate autophagosome generation and initiate the mitophagy pathway. However, there is mitophagic dysfunction in AD [2,17]. Furthermore, the dysfunctional mitochondria in AD may also accumulate in neuronal cells due to the longer lifespan of mitochondria in the brain [13]. 

The purpose of this article is to highlight Aβ- and P-Tau-induced mitochondrial dysfunction, autophagy, and mitophagy in AD. This article also discusses several mechanisms of autophagy and mitophagy in AD. This article also summarizes how the reduced mitochondrial fission protein Drp1 enhances autophagy and mitophagy in AD. 

## 2. Mitochondrial Structure, Function, and Physiology

The mitochondrion is an intracellular organelle responsible for the majority of ATP necessary for mechanisms and cellular pathways with an energy requirement. Mitochondria are also responsible for producing coenzyme A, a critical component of the neurotransmitter acetylcholine, and converting fatty acids to readily available energy sources. Mitochondria also produce many byproducts, including reactive oxygen species (ROS) such as superoxide (O_2_^−^), hydroxyl radical (OH), and hydrogen peroxide (H_2_O_2_) [18]. Furthermore, damage to mitochondria may result in the release of Ca^2+^ and even cytochrome *c*, which can signal cell death [18].

Mitochondria exist in the cell as a network which is beneficial to mitochondrial DNA and structural integrity, as well as overall biogenesis. When a mitochondrion fuses membranes with another to form one large mitochondrion, it is called fusion [19,20]. The reverse—division or fission—results in an increase in mitochondrial number, and a reduction in size [19,20]. Mitochondrial motility is supported by a network of microtubules upon which the organelles are shuttled throughout the cell [21]. Kinesin superfamily proteins (KIFs) and cytoplasmic dynein are the engine and hardware of microtubules that transport mitochondria throughout axons [16]. This movement is critical for neurons, which have long axons requiring mitochondrial transport in order to service their full extent [21]. Mitochondria also suffer damage and dysfunction as they age, owing to oxidative damage, loss of proton gradient, membrane lipid peroxidation, cardiolipin loss, and mitochondrial DNA damage, among other causes. To mitigate the damage to the surrounding organelles and the cell as a whole, mitophagy, a mitochondrial-selective autophagy is performed to eradicate damaged mitochondria [19,20]. Together, fission, fusion, transport, and mitophagy encompass the main processes of mitochondrial dynamics [19,20]. In AD, mitochondrial dynamics are believed to be upset by the phosphorylation of tau proteins, which are structural components of microtubules, or interaction with amyloid beta polypeptides, which amalgamate extracellularly and traverse membranes to interact with mitochondria [22,23]. Furthermore, reduction in biogenesis upsets mitochondrial dynamics because ATP is necessary for transporting mitochondria, maintaining a widespread distribution of mitochondria throughout neuronal cells, and sustaining the rate of transport through the cell [16]. Much of the root of mitochondrial dysfunction actually originates with mitochondrial byproducts of oxidative phosphorylation. 

The foundation of the oxidative phosphorylation pathway begins outside of the mitochondrion with glycolysis. In the cytoplasm of the cell, a molecule of glucose is broken down and phosphorylated to two molecules of glyceraldehyde-3-phosphate, which through a series of phosphorylation, dephosphorylation, and dehydration events are converted to two molecules of pyruvate [24,25]. The pyruvate dehydrogenase complex converts pyruvate to acetyl-CoA via pyruvate decarboxylation [22]. In this form, the two-carbon acetyl group bound to coenzyme A reacts with oxaloacetate to form citric acid in the Krebs cycle, which is progressively oxidized to carbon dioxide through the Krebs cycle [26,27]. This occurs in the matrix of the lumen of the mitochondrion. Through the process of the Krebs cycle, which occurs in the mitochondrial matrix, mitochondria reduce FAD (oxidized form of FADH_2_) to FADH_2_ (flavin adenine dinucleotide) and NAD^+^ to NADH, and the energy needed to drive the electron transport chain (ETC) is stored in the body energy of nitrogen bound to hydrogen in FADH_2_ and in the carbon bound to hydrogen in NADH [26,27].

At the center of biogenesis within the mitochondrion are the complexes I, II, III, IV, and V [28]. Respectively, they are also called NADH-ubiquinone oxidoreductase, succinate dehydrogenase, cytochrome *bc*_1_ complex, COX, and ATP synthase [28,29]. Complexes I, II, IV, and V are transmembrane proteins fitted within the IMM [28]. Complex I oxidizes NADH to NAD^+^, and transports a proton to the intermembrane space between the IMM and the OMM [28,29]. Coenzyme Q_10_ is adjacent to complex II, accepts electrons from complexes I and II, and transfers them along the ETC to complex III [28]. Complex II is peripheral to the IMM, oxidizes FADH_2_ to FAD, and complex III pumps protons across the IMM into the intermembrane space [28,29]. Carrier C, aka cytochrome *c*, transports electrons to complex IV from complex III, and protons pumped by complex IV reduce oxygen to water (H_2_O) [28]. The large proton gradient between the intermembrane space and the matrix provides the proton-motive force that drives complex V’s phosphorylation of ADP to ATP [28]. Unlike the outer membrane, the IMM lacks the cholesterol required to give the membrane fluidity. The IMM does however have cardiolipin—a phospholipid which is targeted and bound by lysine desuccinylase SIRT5, enabling its function [30]. Cardiolipin’s primary role of regulating the electron transport chain is due to its intramolecular bonding and unsaturated fatty acid tails, giving it a conical three-dimensional structure that curves deep folds into the IMM [31,32]. These deep folds project almost diagonal to one another, and tightly pack a large IMM with an expansive surface area, allowing multiple successions of complexes along the membrane to conduct electron transport and oxidative phosphorylation [31,32]. 

The ETC is also responsible for reactive oxygen species (ROS) production. There is an element of inefficiency where electrons escape the electron carriers of the ETC to dioxygen molecules to form superoxide I [33,34]. The guilty parties in this superoxide formation are typically complex III, specifically their ubiquinone sites, and the flavin mononucleotide group of complex I [33,34,35]. Superoxide dismutase in mitochondria rapidly converts O_2_^−^ to H_2_O_2_, another less-damaging ROS, via the dismutation redox reaction [33,34,35]. The ROS production from mitochondria plays an integral role in the cellular aging process, and in the physiological aging of an organism, including the development of diseases [34]. In tissues and systems where mitochondria are needed the most (e.g., cardiac tissue, central nervous system, and hepatic tissue), the ROS damage of mitochondria brings about a plethora of complex dysfunctions such as cardiac ischemia (e.g., ischemic reperfusion [36], cardiomyopathy [37], congenital heart diseases [38]), neurodegenerative diseases (e.g., Huntington’s disease [39], amyotrophic lateral sclerosis [40], Parkinson’s disease [41], Friedreich ataxia [42], Alzheimer’s disease [43]), and chronic liver diseases (e.g., Pearson’s syndrome [44], villus atrophy syndrome [45], neonatal liver failure [46], Alpers–Huttenlocher syndrome [47]).

The cell’s main defense against mitochondrial dysfunction and the ensuing damage is mitophagy—a mitochondria-specific autophagy [48]. Nonetheless, in severely chronic diseases such as AD, mitophagy is also impaired [48]. This is due to changes in the structural conformation of amyloid-beta protein to a disease-causing conformation, phosphorylated tau damage, and resultant cellular and mitochondrial damage [49,50].

## 3. Alzheimer’s Disease and Mitochondrial Dysfunction

Mitochondrial abnormalities, including impaired mitochondrial dynamics (increased fission and reduced fusion), altered mitochondrial morphology, altered mitochondrial gene expressions, increased free radical production and lipid peroxidation, and reduced COX activity and ATP production are largely reported in AD. These mitochondrial abnormalities lead to mitochondrial dysfunction in the progression and pathogenesis of AD [6,10,13,51,52,53,54,55,56,57,58,59,60,61,62,63,64,65,66,67]. We also summarize some seminal and important mitochondrial studies in AD below: 

### 3.1. Mitochondrial Gene Expression Changes and Alzheimer’s Disease

Using global microarray analysis, Reddy and colleagues [52] measured mRNA levels in transgenic APP (amyloid precursor protein) mice at three different ages: 2-, 5-, and 18-months. They found that the genes related to mitochondrial energy metabolism and apoptosis were up-regulated in all three age groups, indicating that energy metabolism is impaired in disease progression of AD. Based on these observations they proposed that mutant APP and soluble Aβ enter mitochondria, induce an excessive production of free radicals, and cause mitochondrial dysfunction. To compensate dysfunctional energy metabolism, mitochondrial-encoded genes were upregulated in APP mice. These findings have important implications for understanding the mechanism of Aβ toxicity in AD. These results are comparable with other research findings. Scheffler et al. found that in AD cells in vivo, Aβ deposition is strongly correlated with mitochondrial-DNA-damaging polymorphisms [68]. Scheffler et al. also found that the removal of Aβ increased ATP production, bioenergetics, and mitochondrial function in microglia cells, which indicates that Aβ accumulation in neuronal cells is a cause for mitochondrial dysfunction [68]. Yan and Stern also found that Aβ reduced the bioenergetics of mitochondria by binding to ABAD and increasing the production of free radicals [69]. Yan and Stern also demonstrated that Aβ interaction with cyclophilin D is responsible for the interference with IMM complexes and reduction in ATP production [69].

In their next paper, Reddy and colleagues [55] tested their hypothesis in a series of experiments examining what forms of APP and Aβ localize to the mitochondria, and whether the presence of these species is associated with mitochondrial dysfunction and oxidative damage. They observed that increased free radicals correlated with soluble Aβ in APP mice. Based on these observations, they proposed that mitochondrial Aβ are likely responsible for increased free radicals in the disease process [52]. These research findings are congruent with the reduction in COX activity found in postmortem AD brains [70]. Mauer et al. concluded that the hippocampus and temporal cortex of postmortem AD brains showed evidence of increased oxidative stress, likely owing to COX deficiency [70]. Furthermore, loss of phospholipids—particularly the cardiolipin of the IMM—exacerbates the pathology of AD [71]. In fact, it is believed that the loss of cardiolipin is greatly responsible for the loss of cytochrome *c* in the IMM [71].

### 3.2. Amyloid Beta Interaction with Drp1 and Increased Mitochondrial Fragmentation

Next, the Reddy group investigated the molecular links between increased mitochondrial fission Drp1 and Aβ using co-immunoprecipitation and colocalization studies. They used postmortem AD brains and brain tissues from APP mice. Drp1 immunoprecipitation/immunoblotting analysis of Aβ antibodies 6E10 and A11 revealed that Drp1 interacts with Aβ monomers and oligomers in AD patients and APP mice, and these abnormal interactions are increased with disease progression. Their colocalization studies using Drp1 and the Aβ antibodies revealed the colocalization of Drp1 and Aβ. These findings suggest that increased production of Aβ and the interaction of Aβ with Drp1 are crucial factors in mitochondrial fragmentation, abnormal mitochondrial dynamics, and synaptic damage in AD [14].

### 3.3. Phosphorylated Tau Interaction with Drp1 and Increased Mitochondrial Fragmentation

In another study, the Reddy lab [72] tested whether P-tau interacts with Drp1, and attempted to elucidate mitochondrial damage in the progression of AD. They also investigated GTPase Drp1 enzymatic activity—which is critical for mitochondrial fragmentation—in postmortem brain tissues from patients with AD as well as brain tissues from three different lines of transgenic APP, APP/PS1, and 3XTg.AD mice. Using co-immunoprecipitation and immunofluorescence analyses, they demonstrated the physical interaction between P-Tau and Drp1 for the first time. Mitochondrial fission-linked GTPase Drp1 activity was significantly elevated in the postmortem frontal cortex tissues from AD patients and cortical tissues from APP, APP/PS1, and 3XTg.AD mice. On the basis of these findings, they concluded that Drp1 interacts with Aβ and P-Tau, likely leading to excessive mitochondrial fragmentation and mitochondrial and synaptic deficiencies, ultimately possibly leading to neuronal damage and cognitive decline [72].

### 3.4. Amyloid Beta Toxicity in Hippocampal APP Neurons and APP Mice Mitophagy

Reddy and colleagues [4] investigated mitochondrial structure and function, as well as autophagy, mitophagy, and synaptic proteins in primary mouse hippocampal (HT22) neurons transfected with mutant APP cDNA (mutant APP). They also studied mitochondrial dynamics, biogenesis, and cell survival in mutant APP cells. Similar to APP mice, they found increased levels of mRNA and proteins of mitochondrial fission genes and decreased levels of fusion, biogenesis, autophagy, mitophagy, synaptic, and dendritic genes in mAPP-HT22 cells relative to WT-HT22 cells. Significantly reduced cell survival was observed in mAPP-HT22 cells. Mitochondrial numbers were increased, and mitochondrial length was significantly reduced in mAPP-HT22 cells. Based on these observations, they concluded that the accumulation of mAPP and Aβ are responsible for abnormal mitochondrial, autophagy, mitophagy, and synaptic activities in mutant APP hippocampal cells. 

In their next study, the Reddy group [73] investigated Aβ-induced mitochondrial, synaptic, autophagy, mitophagy, synaptic activities, and cognitive behavior in 12-month-old APP transgenic mice. Hippocampal learning and memory and motor learning and coordination were impaired in APP mice relative to WT mice. Mitochondrial dynamics were defective, and mitochondrial biogenesis, autophagy, mitophagy, synaptic, and dendritic proteins were significantly reduced in APP mice. Further, dendritic spines were reduced in APP mice. These observations strongly suggest that the hippocampal accumulation of mutant APP and Aβ are responsible for mitochondrial dysfunction and synaptic damage and defective autophagy and mitophagy in APP mice [73]. 

### 3.5. Phosphorylated Tau Toxicity in Hippocampus and Mitophagy—Mutant Tau Mice

In another study, the Reddy lab [74] studied mitochondrial dynamics, biogenesis, and synaptic proteins in the hippocampus of 12-month-old transgenic Tau mice. Mitochondrial dynamics were impaired, meaning increased fission and reduced fusion in Tau mice relative to age-matched WT mice. Interestingly, synaptic and dendritic proteins were also altered in Tau mice, and reduced synaptic and dendritic proteins were correlated with cognitive decline. Based on these observations, they propose that the hippocampal accumulation of phosphorylated tau is responsible for synaptic damage and hippocampal-based learning and memory impairments in tau mice [74]. 

### 3.6. Reduced Drp1 and Protection against Aβ-Induced Toxicities.

Previous studies from the Reddy lab have provided compelling evidence that Aβ and P-tau interact with Drp1 and induce GTPase Drp1 enzymatic activity and fragment mitochondria excessively [14,75,76]. They proposed that a partial loss of Drp1 reduces GTPase Drp1 activity and reduces mitochondrial fragmentation in AD neurons. To test their hypothesis, they crossed Drp1 heterozygote knock-out (Drp1+/−) mice with APP transgenic mice and created double-mutant (APP x Drp1+/−) mice. They extensively studied mitochondrial dynamics, biogenesis, and synaptic activity in 6-month-old Drp1+/−, APP, APP x Drp1+/−, and WT mice. Decreased levels of fission and matrix genes and increased levels of fusion, biogenesis, and synaptic genes were found in 6-month-old double-mutant mice relative to APP mice. Mitochondrial dysfunction was reduced in double-mutant mice, suggesting that reduced Drp1 enhances mitochondrial function in AD neurons. These findings suggest that a partial reduction of Drp1 is protective against Aβ-induced toxicities in APP mice [77].

### 3.7. Reduced Drp1 and Protection of Phosphorylated Tau-Induced Toxicities

Similar to APP mice, they also crossed Drp1+/− mice with Tau transgenic mice and created double-mutant (Tau x Drp1+/−) mice and studied several aspects of mitochondria. Decreased mRNA and protein levels of fission and matrix genes, and increased levels of fusion, mitochondrial biogenesis, and synaptic genes were found in 6-month-old double-mutant mice relative to Tau mice. Mitochondrial dysfunction was reduced in double-mutant mice relative to Tau mice. P-Tau was found to be reduced in double-mutant mice relative to Tau mice [78]. These findings from double-mutant mice together with APP x Drp1+/− mice suggest that a partial reduction of Drp1 is beneficial against P-tau and Aβ toxicities in AD [77,78].

## 4. Autophagy

**Figure 2:**
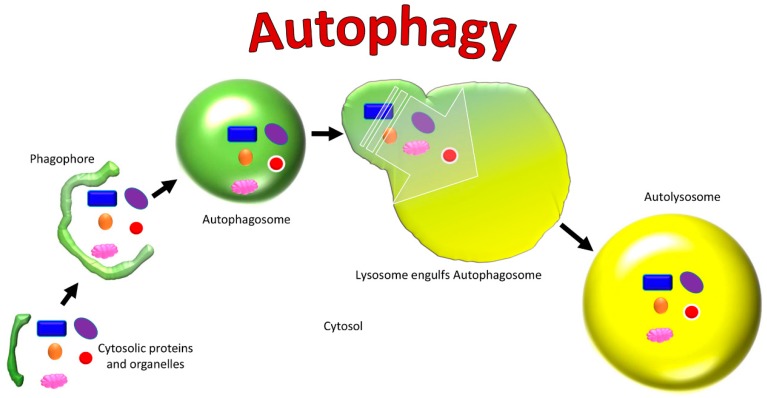
Cellular changes in autophagy.

Autophagy is a process used to maintain balance in healthy cells, organelles, proteins, and nutrients in an organism (Figure 2) [79]. It can be selective or non-selective, depending on whether the targets are specific cellular components or an entire cell [79]. There is macro-autophagy, micro-autophagy, and chaperone-mediated autophagy, which recruit lysosomes and proteolytic enzymes for the degradation of cells and their cytosolic components [79]. The autophagy process begins with specific proteins called autophagy-related proteins (Atg) which cleave to the target and initiate the autophagy cycle [80]. 

**Figure 3:**
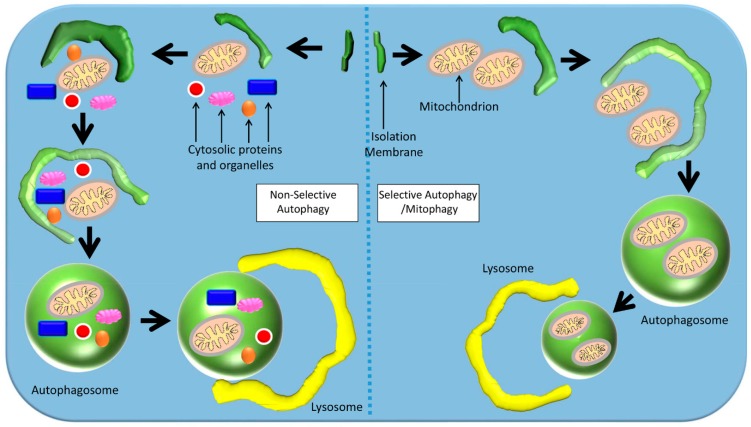
Mechanisms of selective and non-selective autophagy. In non-selective autophagy, a double-membraned autophagosome non-selectively engulfs components of the cytoplasm around its target. In selective autophagy, the autophagosome targets a specific organelle (e.g., mitochondria).

In macro-autophagy, also known as “non-selective autophagy”, a double-membraned vesicle called an autophagosome engulfs its target by sequestering some portion of the cytoplasm, including contents such as organelles (Figure 3) [79,81,82]. The autophagosome membrane then fuses with the lysosome to form an autolysosome, where the contents are destroyed by lysosomal proteases [79,81,82]. In micro-autophagy, via invagination of its membrane the lysosome directly engulfs and degrades cytosolic components itself [79]. It is rare that mitochondria are engulfed with cytoplasm in non-selective autophagy, though when this occurs it is evidence for non-selective autophagy [80]. 

Triggers for autophagy are numerous, and often center on mitochondria [82]. These include the release of cytochrome *c*, the opening of the mitochondrial permeability transition pore (mPTP), increased ROS, and oxidative damage evident from hydrogen peroxide, superoxide, and 2-methoxyestradiol [82]. The initial response to mitochondrial dysfunction would be selective autophagy of damaged mitochondria, which is called mitophagy [82]. Overwhelming dysfunction would cause for autophagy at grander scales, and apoptosis [82].

### 4.1. Selective Autophagy

Selective autophagy is able to target specific tissues, malignant cells, damaged organelles, aggregated proteins, invasive pathogens, and peroxisomes in excess (Figure 3) [83]. Selective autophagy is also involved in the regulation of free iron by regulating the concentration of the iron-chelating protein ferritin [83]. Here, a specific target is taken up by an autophagosome, and non-targets are excluded [81,83]. This is achieved by the use of a nascent autophagosome’s cargo receptor proteins, which attach to the targeted cargo via ATG8-family proteins on the isolation membrane [83]. The cargo receptor proteins recognize poly-ubiquitin chains on the surface of the targeted cargo, which triggers selective autophagy [83]. In chaperone-mediated autophagy (CMA, a form of selective autophagy), chaperone proteins such as Hsc-70 transport target proteins to lysosomal receptors such as lysosomal-associated membrane protein 2A (LAMP-2A), and then across the lysosomal membrane into the lysosome [79].

### 4.2. Mitophagy

**Figure 4:**
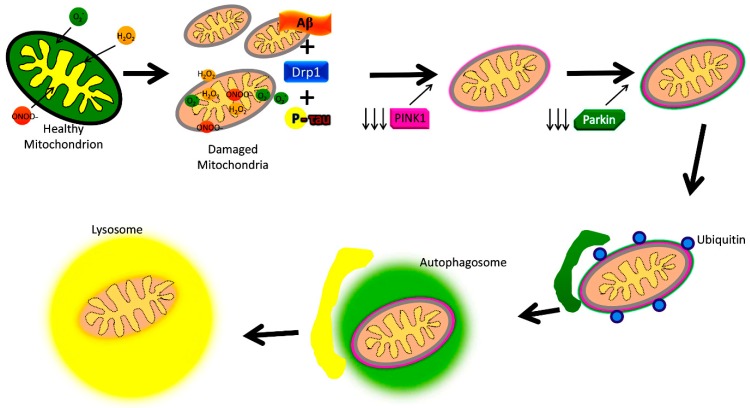
Amyloid beta and phosphorylation-induced defective autophagy and mitophagy in Alzheimer’s disease.

The best-understood pathway for mitophagy is the PTEN-induced putative kinase1 (PINK1) and parkin-induced mitophagy pathway (Figure 4) [2]. 

Increased levels of Aβ and P-Tau, and abnormal interactions between Aβ and Drp1, P-Tau, and Drp1 inducing increased mitochondrial fragmentation and reduced mitochondrial fusion are extensively reported in AD. These abnormal interactions result in the proliferation of dysfunctional mitochondria in AD neurons. Depleted parkin and PINK1 levels occur due to increased accumulation of Aβ in the cytoplasm, reducing the effective number of autophagosomes targeting dysfunctional mitochondria. Dysfunctional lysosomes with reduced capacity are also evident within AD neurons, which contributes to the amassing of proteolytic substrates in defective neurons. Dysfunction in mitophagy is often a cause for worsening disease pathology in AD, where mitochondrial dysfunction plays a central role in pathogenesis.

The two main proteins PINK1 and the ubiquitin ligase protein parkin are responsible for this pathway’s initiation [7,13,84,85,86]. One effect of damage to a mitochondrion is the depolarization of the IMM [84]. This leads the surface receptor protein PINK1 to change to its stable conformation and bind to the OMM [54]. This action then attracts parkin to the OMM, which then begins E3 ligase activity in the cytoplasm [7,84,85,86]. Parkin induces ubiquitination and conformational changes of OMM proteins, labeling them to be recognized and bound by optineurin (OPTN), p62, NDP52, and NBR1, which shuttle the mitochondrion along the mitophagy pathway [7,84,85,86]. The final steps involve engulfment by an autophagosome, which then fuses membranes with a lysosome, destroying the mitochondrion [7,14,84,85]. This particular mitophagy pathway is descriptive of stress-induced mitophagy [86]. Mitophagy may be categorized as non-selective or selective, and furthermore as basal, stress-induced, or programmed mitophagy [86].

### 4.3. Basal Mitophagy

This form of mitophagy is likely the least studied and least understood, since most studies of mitophagy have been with in vitro cell cultures placed under experimentally induced stress, which were analyzed for overexpressed components [86]. Nonetheless, it is believed that mitophagy is a process integral to normal mitochondrial network homeostasis, where a basal quantity and quality of mitochondria are sustained [86]. In fact, the rate of mitophagy varies by tissue type, suggesting a plausible autonomous control of basal mitophagy by different cell types [86]. Compared to stress-induced mitophagy, basal mitophagy is governed independent of PINK1, and likely parkin as well [87,88]. In experimentation with Pink1 KO mouse tissues expressing the mito-QC transgene, basal mitophagy was able to occur without the presence of PINK1 [87]. In experimentation with *Drosophila*, Lee et al. noted that there was no decrease in mitophagy in flies absent of parkin or PINK1 [88].

### 4.4. Stress-Induced Mitophagy

Internal metabolic dysfunction and external factors trigger stress-induced mitophagy [80]. Mitochondrial uncoupling, including uncoupling proteins like UCP2, induce the PINK1–parkin activities of stress-induced mitophagy [86,89]. Nitrogen starvation and/or oxygen deprivation induce receptor-mediated mitophagy [80]. As in other forms of mitophagy, stress-induced mitophagy manages the quality and quantity of mitochondria within the cell, though specifically responding to starvation [80]. Under stressed conditions, respiration continues increasing oxidative damage and escalating Atg32 levels, which anchor to mitochondria to usher in the mitophagy pathway, and likely also monitor the pace of the pathway [80]. The pro-apoptotic Bcl-2 family of proteins also begin to outnumber the anti-apoptotic Bcl-2 proteins, and the mitochondrial membrane integrity begins to wane and increase in permeability, becoming more polarized [90]. The depolarization of the membrane and opening of pores would allow for the release of cytochrome *c*, leading to apoptosis, and thus the mitophagy pathway must intervene to eliminate the dysfunctional mitochondrion and not the entire cell [90]. Instead, parkin/PINK1-regulated mitophagy is initiated, where ubiquitination attracts mitophagy-specific proteins to the target mitochondrion [90]. In addition, stress-induced mitophagy utilizes proteins Atg13, Atg17, Atg29, and Atg31, as noted by Eiyama et al., where in their experiments mitophagy was inhibited under stressed conditions in yeast cells lacking these proteins [80]. A scaffold of proteins Atg17, Atg29, and Atg31 is formed, which allows for the complex of Atg1 through Atg13 protein kinases to propagate the mitophagy pathway—particularly the lipid membranes which form the autophagosome around the mitochondrion [80].

### 4.5. Programmed Mitophagy

As a necessary process during development, mitophagy also occurs at planned intervals during development to clear mitochondria from specific cell types [91]. For example, NIP3-like protein X (NIX)-mediated mitophagy is necessary for the removal of mitochondria during erythropoiesis [92]. Programmed mitophagy is also needed for cell differentiation during retinal ganglion cell development [93]. In fact, this form of mitophagy is used to change from oxidative phosphorylation to glycolysis as the main source of ATP, which propagates the differentiation of retinal ganglionic cells [93]. In this example, in order for differentiation to occur, Atg5 and NIX proteins usher mitochondria along the mitophagy pathway [93]. During the maturation of cardiomyocytes, mitochondria which primarily use glucose for ATP are replaced using with mitochondria which preferentially use fatty acids through programmed mitophagy [94]. Programmed mitophagy plays key roles in the specified development of cells, tissues, and organs throughout the body. It is believed that the dysfunction of programmed mitophagy results in degeneration—particularly neurodegeneration, accelerated aging, mutations, and cancer [91].

### 4.6. Mitophagy Regulation

There are many protein components which regulate the mitophagy pathway (Figure 4). Mitochondrial fission often precedes mitophagy, and is regulated by the proteins Drp1, Fis1, Mff, and the related proteins MiD49 and MiD51 [95]. Mitochondrial fusion is regulated by the outer membrane mitofusin proteins Mfn1 and Mfn2, and the optic atrophy 1 (OPA1) protein of the IMM membrane [6]. Fis1, OPA1, Drp1, Mfn1, and Mfn2 also have integral roles in the mitophagy process, though all aforementioned proteins contribute indirectly. For instance, Mff recruits Drp1 for fission, while MiD49 and MiD51 also bind to Drp1 and may affect fission [95]. 

As shown in Figure 4, mitophagy is defective in AD because of interactions between P-Tau and Aβ with mitochondria, leading to altered expression of autophagy/mitophagy-associated proteins. Details of these autophagy and mitophagy proteins in both AD and healthy states are given below. 

### 4.7. Fis1

**Figure 5:**
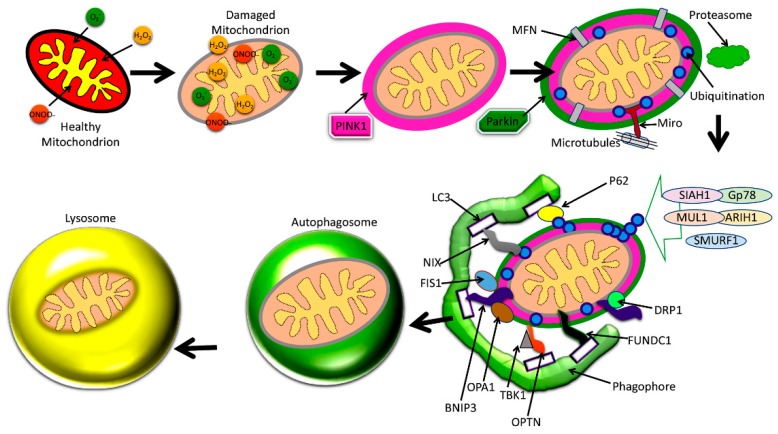
Proteins involved in autophagy and mitophagy in a cell. Figure depicts overall autophagy and mitophagy events and proteins.

Under stressed conditions, Fis1 contributes to the fission of mitochondria which are en-route to mitophagy (Figure 5) [95]. Shen et al. demonstrated that Fis1 likely contributes to the mitophagy pathway, as the inhibition of Fis1 suppresses and retards the mitophagy process, and even results in LGG-1/LC3-1 aggregates, while overexpression of Fis1 increases autophagy [95].

### 4.8. OPA1

OPA-1 is responsible for fusion of the inner membrane and upkeep of mitochondrial cristae structure [96]. OPA-1 is broken down by IMM zinc metalloprotease (OMA1), and various “ATPases associated with diverse cellular activities” (AAA) proteases. It was also demonstrated that Aβ42 downregulates OPA1 [97]. Aβ-42 induces neuronal apoptosis by targeting mitochondria [97].

### 4.9. PINK1

PINK1 initiates DRP1 activity by an indirect route to induce the fission of dysfunctional mitochondria, which enables them to be degraded via autophagy [86]. PINK1 adds phosphoryl groups to ubiquitin and polyubiquitin chains on dysfunctional mitochondria, and it is believed that this labels the mitochondria as a target for engulfment and degradation [86]. After PINK1 is shuttled to the IMM, it is processed, and various proteases cleave PINK1 which is soon degraded [86].

### 4.10. Ubiquitin

Ubiquitin (Ub) is a protein used to mark cargo as a target for autophagy [98]. During mitophagy, it is phosphorylated by PINK1, which phosphorylates the S65 domain of Ub via the serine/threonine of its active site [98]. This effectively induces allosteric activation of parkin and recruits autophagic proteins, receptors, and other autophagic biomolecules [98].

### 4.11. Parkin

Parkin, an E3 Ub ligase, is responsible for the ubiquitination of mitochondrial OMM proteins, which allows them to be recognized for autophagy [86,98]. It is believed that parkin-mediated ubiquitination may also be tissue- or cell-specific [86].

### 4.12. BNIP3

Bcl-2 nineteen-kilodalton interacting protein 3 (BNIP3) is a BH3-protein that induces autophagy and mitophagy, and has an integral impact on cell death and mitochondrial dysfunction (Figure 5) [82]. BNIP3 plays a critical pro-apoptotic role in autophagy, and the dysregulation of its translation is closely linked to the development of cancers as well as the deregulation of mitophagy, autophagy, and cell death [82]. BNIP3 localizes to mitochondria within the cell, induces mitochondrial dysfunction, and promotes the release of apoptotic factors, leading to cell death [82]. Thus, upregulation of BNIP3 in particular causes abnormal reduction in mitochondria and cell death [82]. 

### 4.13. LC3

Microtubule-associated protein 1A/1B-light chain 3 (LC3-1) is pervasive within mammalian tissues and even in-vitro cells, and plays an integral role in the autophagy pathway [99]. LC3-1, which is relatively small in size (~17kDa), is converted in the cytoplasm to form the LC3–phosphatidylethanolamine conjugate (LC3-II), which attaches to and recruits autophagosome membranes to a target [82]. LC3-II proliferation often result from starvation-induced autophagy.

### 4.14. NIX

A homologue of BNIP3, NIX/BINP3L, also induces mitophagy and is especially important for the removal of maturing red blood cells [82]. Evidence shows that NIX interacts directly with and binds to LC3-1, which tethers the mitochondrion to the autophagosome [82].

### 4.15. OPTN

Optineurin (OPTN) along with NDP52 (CALCOCO2), TAX1BP1, and p62 are utilized during mitophagy to adhere mitochondria and ubiquitinated proteins to autophagic membranes [98]. Along with p62, OPTN is phosphorylated by protein kinases marking autophagic membranes or poly-ubiquitinated cargo to initiate autophagy [98]. OPTN’s TAX1BP1 and SKICH NDP52 domains are phosphorylated by TBK1 [98].

### 4.16. FUNDC1

FUN14 domain-containing protein 1 (FUNDC1) is a mitochondrial OMM protein that plays an essential role in mitophagy induced by oxygen deprivation [100]. FUNDC1 is a highly conserved protein, which localizes to mitochondria and binds to LC3-1, encouraging the mitochondrial autophagy pathway [100].

### 4.17. TBK1

TANK-binding kinase 1 (TBK1) functions by activating OPTN through phosphorylation of its S513 domain, increasing the retention of the OPTN S473 domain to increase OPTN’s binding capacity to additional ubiquitin polypeptides, and phosphorylates the S177 domain to increase LC3-1 binding affinity [98].

### 4.18. SIAH1

Seven in absentia homolog 1 (SIAH1) regulates the α-synuclein monoubiquitination, and induces α-synuclein accumulation and intracellular inclusion formation [101]. SIAH1 activity is essential for inducing the autophagy degradation pathway [101].

### 4.19. MUL1

Mitochondrial E3 ubiquitin protein ligase 1 is an E3 ubiquitin protein ligase that mediates ATG5- and ULK1-dependent mitophagy initiated by selenite [102].

### 4.20. ARIH1

Ariadne RBR E3 ubiquitin protein ligase 1 (ARIH1) is an E3 ubiquitin protein ligase that targets proteins for autophagy via ubiquitination [103]. It has the ability to polyubiquitinate damaged mitochondria, and its activity is especially important for resistance to cancer growth [103].

### 4.21. SMURF1

The Smad ubiquitin regulatory factor 1 (SMURF1) protein is an E3 ubiquitin ligase that plays the essential role of mediating the ubiquitination of target substrates for proteases to break down during autophagy [104].

### 4.22. Gp78

Glycoprotein 78 (Gp78) plays an important role in endoplasmic-reticulum (ER)-associated degradation, which acts to transfer ubiquitin from an E2 ubiquitin-conjugating enzyme to a protein substrate—an essential step in the ubiquitination of cargo receptor proteins during autophagy (Figure 5) [105]. Upon loss of mitochondrial membrane potential, Gp78 is activated to initiate mitophagy and attract enhanced green fluorescent protein (EGFP)-LC3-1—an autophagosome marker—to the mitochondria-associated ER [105]. Gp78 anchors to the ER and initiates the degradation of mitochondrial mitofusin Mfn1 and Mfn2 proteins for degradation, while inducing mitochondrial fragmentation [105]. It is also involved in the degradation of CD3-delta T-cell receptor, ApoB lipoprotein, HMG CoA reductase, cystic fibrosis transmembrane conductance regulator, and the metastasis suppressor KAI1 [105]. 

### 4.23. Proteasome

A proteasome is a complex of proteases, which are enzymes with active sites oriented to denature and degrade proteins, specifically marked for degradation by ubiquitination (Figure 5) [106]. In fact, 80–90% of proteins are degraded via the ubiquitin–proteasome system [106]. The most prolific proteasome is 26 S, which is composed of the central 20 S proteasome and the 19 S regulatory particle, which are about 700 kDa and 900 kDa, respectively [106].

### 4.24. MIRO

Mitochondrial rho (MIRO) is a Ca^2+^ sensor that controls mitochondrial transport with Ca^2+^ dependency [16]. MIRO forms a complex with KIF5, and Milton (or KIF5), and TRAK2 (an orthologue of Milton)—both motor proteins—to shuttle mitochondria through the dendrites and axons of hippocampal neurons or cardiomyocytes [16]. MIRO is a mitochondrial OMM protein [107]. During autophagy, its degradation is triggered by the phosphorylation and activation of PINK1 and parkin, which initiates the excision of the mitochondrion via mitophagy [107]. Note that dysfunction of the MIRO protein also inhibits mitochondrial transport, and without the ability for mitochondria to approach one another to fuse, induces mitochondrial fragmentation [16]. 

### 4.25. MFNs

Mitofusin, or MFN, proteins are essential to mitochondrial dynamics, and operate to induce mitochondrial fusion [73,105]. Mfn1 and Mfn2 are GTPases that localize to the OMM and bind GTP at the N-terminals and transmembrane proteins at the C-terminals of their catalytic domains [73]. During autophagy, both Mfn1 and Mfn2 depend on Gp78 for ubiquitination and subsequent degradation [62].

### 4.26. Phagophore

Phagophores are formed from doubly membraned enclosures encircling portions of cytoplasm [108]. Once formed, the hemispherical “cup”-shaped phagophore will enclose and fully engulf a portion of the cytoplasm during autophagy, and the newly formed vesicle is termed an autophagosome [108]. The source of membrane for the phagophore is the Golgi apparatus, which assists with lipids and proteins for the phagophore to fully develop into an autophagosome [108].

### 4.27. P62

Along with OPTN, p62 is phosphorylated by protein kinases marking autophagic membranes or poly-ubiquitinated cargo to initiate autophagy [98]. TBKl regulates phosphorylation of the UBA and LIR domains of p62 for the autophagy pathway as well [99].

## 5. Defective Mitophagy and Alzheimer’s Disease

Mitophagy dysfunction may happen normally with aging, but is worsened by AD [6,109,110,111]. Neuronal mitochondria’s lifespan assists in the worsening of AD pathology when combined with the impediments to mitophagy [2,78]. Mitophagy dysfunction includes accumulation of excessively large autophagic vacuoles (AVs) in the cell body of neurons, especially within dysfunctional neuritis [13]. AVs are often filled with Aβ peptides, and become an opportune location for Aβ to accumulate [13]. It is believed that functional PS1 protein is also necessary for the completion of lysosomal maturation, but the AD-related mutations in PS1 which lead to the development of early-onset AD also impair the lysosomal degradation of defective mitochondria in the mitophagy pathway [2]. AD is also related to MIRO mutation, and dysfunction with this protein necessary for the mitophagy pathway is related to Aβ_42_-connected AD development [2]. The most impactful damage of AD to mitophagy is the reduction of parkin and PINK1 proteins, which reduces the amount of successful mitophagy pathways and increases the amount of dysfunctional mitochondria [76]. This also contributes to dysfunctional mitochondrial transport, increased hyperphosphorylation of tau, and synaptic dysfunction [75].

In addition, the spatial dynamics of neuronal mitochondria play an essential role in how autophagy dysfunction exacerbates or possibly induces AD symptoms. In a study by Cai et al. (2012), it was noted that neuronal mitophagy is a much slower process than the mitophagy of somatic cells, particularly due to the concentration of mature lysosomes in the cell body, despite mitochondria covering the vast expanse of dendrites and axons of neurons [112]. Their study controlled for parkin-mediated mitophagy by eliminating parkin, and noted the slower rates of mitophagy of depolarized (dysfunctional) mitochondria [112]. Here, impaired transport induced mitophagy dysfunction, and vice versa [112].

In a study by Tammineni et al. (2017), they studied Aβ peptides and Aβ oligomers (AβOs) in mutant hAPP mice and AD patient cells, and it was found that AβOs interacted with autophagic vacuoles in the distal axons of mutant mouse cells [113]. In fact, this association with autophagic vacuoles was responsible for dysfunction within the axon, particularly with amphisomes and inhibition of mitochondrial axonal transport, where the two dysfunctions worsen the effects of each other [113]. 

Another study by Guglielmotto et al. (2014) attested that intracellular Aβ monomers, rather than oligomers, impacted autophagy to a greater extent [114]. Guglielmotto’s lab noted that AβO caused augmentation of LC3-II, and beclin translation affected dynamics of autophagy, but not nearly as close to the degree that Aβ monomers inhibited the pathway of the endosome-lysosomal machinery [114].

## 6. Mitochondrial Dysfunction and Mitophagy Impairment in Alzheimer’s Disease

Mitochondrial dysfunction is part of the routine mitochondrial dynamic cycle, and is a central proponent of the mitophagy pathway (Figure 4) [2,13]. Mitochondria may undergo fusion and increase in size, while reducing their numbers; or undergo fission and reduce their size, while increasing in number [6,76,115]. Through fusion, essential resources may amalgamate and benefit the health of mitochondria by becoming one, and through fission, mitochondria may increase in number, and dysfunctional mass may be excised [6,115]. Mitochondrial transport is another component of mitochondrial dynamics, where mitochondria may exhibit motility throughout the cell via a network of microtubules in cytoplasm [13]. Mitochondrial transport is especially necessary in neuronal cells, where biogenesis along the long axons and dendrites is only possible via mitochondrial axonal transport [13,76]. Mitochondrial dysfunction does necessitate selective autophagy (i.e., mitophagy), and it completes the cycle of mitochondrial dynamics [2]. Factors such as oxidative damage, cytochrome *c* leakage from the IMM, Ca^2+^ release, loss of membrane potential, caspase activation, and mtDNA damage are signs of mitochondrial dysfunction, and precede selective autophagy [2,13,76,116]. With mitophagy, damaged mitochondria are excised from the mitochondrial network, increasing the ratio of healthy mitochondria and biogenesis vs. dysfunctional mitochondria and oxidative damage. Further, it should be noted that the coding gene poly-(ADP-ribose)-polymerase 1 (or *PARP*1) and the sirtuin SIRT1 utilize the same resource (i.e., NAD^+^, a coenzyme essential to their function), and likely inhibit one another [117]. This results in a limitation to biogenesis, and mitochondrial DNA damage with age, which further exacerbates mitophagy dysfunction [117]. Mitophagy dysfunction is a trigger for apoptosis, where neuronal death is a component of AD pathology [117].

Mitochondrial quality control is maintained through balanced mitochondrial dynamics [13,76,116]. Aberrant autophagy in AD cells is evidenced by the proliferation of dysfunctional mitochondria, often losing structural integrity, and visibly misshapen [13]. This may be attributed to depleted parkin and PINK1 levels due to increased accumulation of Aβ in the cytoplasm, reducing the effective number of autophagosomes targeting dysfunctional mitochondria [13]. Dysfunctional lysosomes are also evident within AD neurons, and have reduced capacity, which contributes to the amassing of proteolytic substrates in defective neurons [13]. Dysfunction in mitophagy is often a cause or means of worsening disease pathology in diseases such as AD, where mitochondrial dysfunction plays a central role in the pathogenesis [2,13,116]. In Parkinson’s disease and AD, for example, there is a loss of PINK1 and parkin function, which deregulates the ability to excise dysfunctional mitochondria and toxic proteins [2,13,116]. 

## 7. Conclusions and Future Directions

Alzheimer’s disease is a multi-factorial progressive neurodegenerative disease with no cure. Tremendous progress has been made in understanding (1) the molecular basis of the disease, (2) biomarker development, and (3) drug discovery. Although much progress has been made and improved our basic understanding, we still do not have a drug and/or agent that can delay and/or prevent dementia in elderly individuals and patients with AD. 

Mitochondrial abnormalities (increased mtDNA damage, low synaptic ATP, increased oxidative stress), synaptic damage, and microRNA regulation and defective autophagy and mitophagy are major cellular events that occur in AD disease development, progression, and pathogenesis, in addition to the accumulation of Aβ and P-Tau. 

Defective autophagy and mitophagy are emerging cellular events that occur due to abnormal interaction between Aβ and Drp1, P-Tau, and Drp1. These interactions lead to increased mitochondrial fragmentation and affect several critical proteins involved in mitophagy (PINK1, parkin, P62, BNIP3, FUNDC1, LC3-1, OPTN, TBK1), autophagy (ATG proteins, LC3-I and II), and ubiquitination (SIAH1, Gp78, MUL1, ARIH1, SMURF1). 

In terms of rescuing and enhancing autophagy and mitophagy, reduced Drp1 and Aβ and P-tau levels and enhancing the levels of PINK1/parkin are proposed to rescue and/or maintain mitophagy and autophagy in affected AD neurons. The continuous clearance of cellular and mitochondrial debris is important for normal cellular function. We need more research on autophagy and mitophagy mechanisms and therapeutic aspects using cell cultures, animal models, and human AD clinical trials.
